# Molecular transmission networks and pre-treatment drug resistance among individuals with acute HIV-1 infection in Baoding, China

**DOI:** 10.1371/journal.pone.0260670

**Published:** 2021-12-02

**Authors:** Penghui Shi, Zhixia Chen, Juan Meng, Miaomiao Su, Xuegang Yang, Weiguang Fan, Haoxi Shi, Ying Gao, Xinli Lu

**Affiliations:** 1 Clinical Laboratory, The People’s Hospital of Baoding, Baoding, Hebei, China; 2 Infection Division, The People’s Hospital of Baoding, Baoding, Hebei, China; 3 Department of AIDS Research, Hebei Provincial Center for Disease Control and Prevention, Shjiazhuang, Hebei, China; University of Malaya, MALAYSIA

## Abstract

**Background:**

Human immunodeficiency virus type 1 (HIV-1) genetic diversity and pre-treatment drug resistance (PDR) are major barriers to successful antiretroviral therapy (ART). In China, sexual intercourse is the most frequent route of HIV-1 transmission. However, few studies have analyzed PDR and transmission networks in detail among individuals in China with acute HIV-1 infection and their sexual contacts.

**Methods:**

A cross-sectional study was conducted in Baoding City, Hebei Province, China from 2019–2020. CD4 T cell counts and viral loads were assessed and a HIV-1 genotypic PDR assay was developed in-house. Transmission networks were visualized using Cytoscape with a threshold genetic distance of 0.015 among HIV-1 subtypes.

**Results:**

From 139 newly diagnosed and drug-naïve individuals with HIV-1, 132 *pol* gene sequences were obtained and revealed eight HIV-1 subtypes. Circulating recombinant form (CRF)01_AE was the most frequent subtype (53.0%, 70/132) followed by CRF07_BC (26.5%, 35/132), B (13.6%, 18/132), unique recombinant forms (2.3%, 3/132), CRF55_01B (1.5%, 2/132), CRF103_01B (1.5%, 2/132), CRF65_cpx (0.8%, 1/132), and C (0.8%, 1/132). A total of 47 *pol* gene sequences were used to generate 10 molecular transmission networks. The overall prevalence of PDR was 7.6% and that of PDR to non-nucleotide reverse transcriptase inhibitors was 6.1%. Of three transmission networks for PDR, two were closely associated with Beijing and Tianjin, while another was restricted to sequences determined in this study.

**Conclusions:**

These results demonstrate that during acute HIV-1 infection, PDR is transmitted in dynamic networks. This suggests that early detection, diagnosis, surveillance, and treatment are critical to effectively control HIV-1 spread.

## Introduction

The Joint United Nations Programme on human immunodeficiency virus (HIV)/acquired immune deficient syndrome (AIDS) (UNAIDS) report showed that 37.7 million people were living with HIV worldwide in 2020 [1]. Over the past four decades, HIV control efforts have been very successful. The number of new HIV infections declined from 2.1 million in 2010 to 1.5 million in 2020, a 31% decrease. Moreover, 1.9 million HIV-positive individuals died from diseases related to AIDS in 2004, while AIDS-related deaths were reduced to 680,000 in 2020 [1]. The success of HIV control has been mostly attributed to the development of antiretroviral therapy (ART) [2–5]. To end the HIV public health threat worldwide by 2030, UNAIDS formulated a 90-90-90 target for 2020 [6] that was included in China’s 13^th^ five-year plan for HIV prevention and control (2016–2020). Two of the three 90s were closely associated with ART (90% of individuals with laboratory-confirmed HIV-1 status receiving sustained ART and 90% of those receiving ART achieving viral suppression).

From its development in 1996 until the end of 2020, 27.5 million HIV-positive individuals worldwide had received ART, while treatment coverage and viral suppressions rates had reached 84% and 90%, respectively [1]. Although ART has effectively slowed HIV transmission, the frequency of mutations leading to drug resistance is increasing with extended use of ART. Pre-treatment drug resistance (PDR) is of special concern. Some studies [7–9] indicated that the prevalence of PDR among HIV-1 isolates exceeded 10% in some regions and may surpass 20% in some counties in southern Africa. A report released by the World Health Organization (WHO) stated that the prevalence of PDR to non-nucleotide reverse transcriptase inhibitors (NNRTIs) was above 10% in 12 of 18 countries surveyed [8]. Thus, the WHO initiated a global strategy for surveillance of HIV drug resistance, including monitoring of PDR. In response to high levels of PDR to NNRTIs, the WHO renewed its guidelines for ART in 2019 to recommend preferential use of non-NNRTI-containing regimens.

In China, the National Free Anti-HIV Treatment Program has operated successfully for 18 years. However, following global trends, the prevalence of HIV-1 strains with PDR increased from 3.7% in 2012 to 6.8% in 2017 [10]. The prevalence of PDR has reached higher levels in some areas: 17.4% in Shanghai [11], 11.5% in Tianjin [12], 12.2% in Liangshan, Sichuan Province [10], 9.3% in Dehong, Yunnan Province, and 8.9% in Lincang, Yunnan Province [10]. These data suggest that region-specific investigations of HIV-1 PDR are required.

Hebei, with an area of 190,000 km^2^, is a province in North China that surrounds Tianjin and Beijing. Hebei is an inevitable route for travel into Beijing and Tianjin. At the end of October 2020, a total of 15,178 individuals with HIV-1/AIDS were living in the province [13]. Of 11 cities in Hebei, Baoding City shares a border with Beijing and Tianjin and was the second most severely affected by HIV-1. HIV-1 infections in Baoding and Shijiazhuang accounted for 37.1% of the provincial total [14]. Baoding plays a key role in disease prevention and control efforts because it adjoins the nation’s capital. However, few studies have analyzed PDR and transmission networks in detail among individuals with acute HIV-1 infection and their sexual contacts in Baoding. To thoroughly evaluate HIV-1 transmission and PDR, a cross-sectional study was conducted in Baoding City.

## Materials and methods

### Ethics statement

Written informed consent was obtained from all adult patients and children’s guardians prior to blood collection. The study was approved by the Medical Ethics Committee of the People’s Hospital of Baoding. All experimental methods and study procedures were performed in accordance with approved regulations and guidelines. The study protocol was approved by the institutional review board of the People’s Hospital of Baoding. The protocol number was 2019–03.

### Study participants

A total of 139 HIV-1-infected individuals were recruited in Baoding City prior to ART initiation during December 2019—December 2020. All participants were newly diagnosed with HIV-1 by western blotting from December 2019 –December 2020. Among these participants, plasma samples from 132 individuals were successfully sequenced and the remaining 7 samples were not amplified because of poor sample quality. All participants had acute HIV-1 infection based on the following national criteria [15]: (1) anti-HIV-1 positive by western blotting, (2) viremia with viral loads (VLs) of 2.17×10^3^–5.54×10^6^ copies/mL, and (3) clinical phase I–IV symptoms [16] such as fever, malaise, herpes zoster virus infection, and persistent diarrhea. Baseline information including demographic and behavioral characteristics were obtained from all participants through face-to-face interviews. Beijing`s sequences and Tianjin`s sequences were downloaded from the HIV Database (http://www.hiv.lanl.gov/content/index).

### Laboratory tests

During December 2019—December 2020, below laboratory tests were carried out. CD4 T cell counts were determined from 50 μL of whole blood (FACS Count; Becton–Dickinson, Franklin Lakes, NJ, USA). The remaining whole blood sample was centrifuged at 3000 rpm to produce blood plasma for HIV-1 VL and PDR assays. HIV-1 VL was quantified using an Amplicor HIV-1 monitor test (COBAS TaqMan 48; Roche, Switzerland). VLs were expressed in log RNA copies/mL. A HIV-1 genotypic PDR assay was implemented as previously described [17] using an in-house method. HIV-1 PDR mutation analysis was performed based on a fragment of the HIV-1 *pol* gene (1.3 kb, HXB2:2147–3462) using the Stanford University HIV DR Database online sequence analysis tool (http://hivdb.stanford.edu/). According to the WHO-recommended criteria for PDR, resistance was classified as low, intermediate, or high resistance for five NNRTIs, two nucleotide reverse transcriptase inhibitors (NRTIs) and one proteinase inhibitor (PI).

### Sequence analysis

Raw sequences were assembled using Contig Express 9.1. Multiple sequence alignment with ClustalW and manual editing were performed using Bio-Edit 7.0 software. A neighbor-joining phylogenetic tree was constructed using the Kimura two-parameter model with 1000 bootstrap replicates in MEGA 6.0. HIV-1 subtypes were identified using the REGA HIV-1 Subtyping Tool 3.0. URFs were determined using the online jumping profile Hiden Markov Model (jpHMM). To construct HIV-1 molecular transmission networks, pairwise Tamura–Nei 93 (TN93) genetic distances were calculated among sequences using HYPHY2.2.4. According to *Technical Guidelines for Monitoring and Intervention of HIV Transmission Network (Trial)* released by Chinese Center for Disease Control and Prevention, Transmission networks were visualized using Cytoscape v3.8.0 with a threshold genetic distance of 0.015 among HIV-1 subtypes.

### Statistical analysis

Statistical analysis was conducted using SPSS 21.0 (SPSS Inc. Chicago, IL, USA). Means or frequencies were used to summarize demographic data. Differences in categorical variables were assessed using the chi-square test. All tests were two-tailed and P-values < 0.05 were considered statistically significant.

## Results

### Demographic and epidemiologic characteristics of subjects

**Fig 1:**
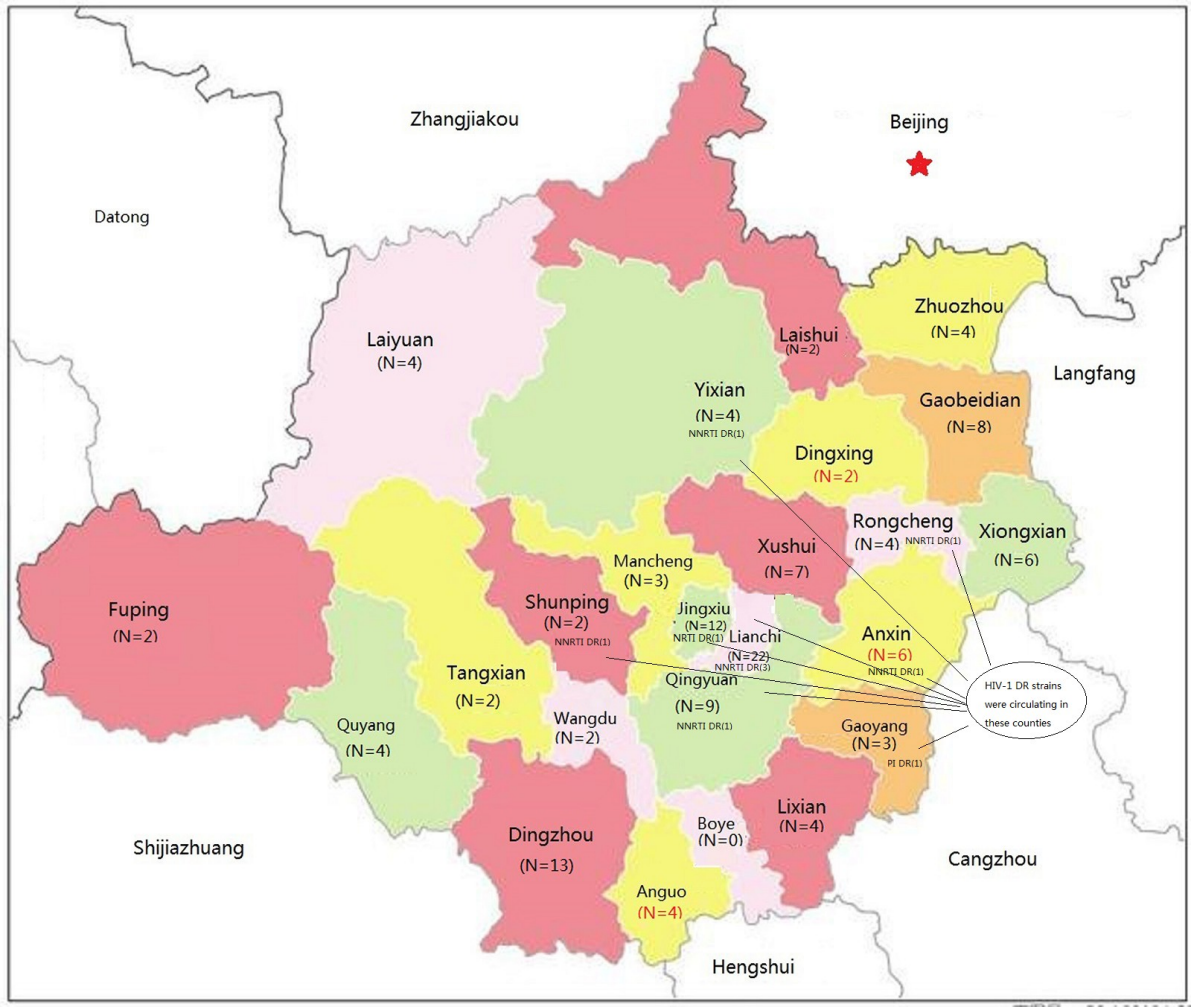
Geographic distribution of participants with acute HIV-1 infection in Baoding. Note: DR, drug resistance; PI, protease inhibitor; NRTI, nucleoside reverse transcriptase inhibitor; NNRTI, non-nucleoside reverse transcriptase inhibitor. This figure was adapted from open access map: http://map.ps123.net/china/14.html with Microsoft PowerPoint 2016.

As shown in Table 1, a total of 132 HIV-1 partial *pol* gene sequences (1.3 kb) were obtained. These 132 HIV-1 individuals resided in 24 of 25 counties in Baoding City, Hebei (Fig 1). Among participants with HIV-1 *pol* sequences, 90.9% (120/132) were male and 9.1% (12/132) were female. The most common age strata were 18–49 years (79.5%, 105/132), ≥50 years (19.7%, 26/132), and <18 years (0.8%, 1/132). The most common transmission routes were transmission among men who have sex with men (MSM) (84.8%, 112/132), heterosexual (HET) transmission (13.6%, 18/132), and other (1.5%, 2/132). Married, unmarried, and divorced/widowed individuals accounted for 56.8% (75/132), 31.1% (41/132), and 12.1% (16/132) of participants, respectively. The most common initial CD4 T cell counts were 200–499 cells/μL (54.5%, 72/132), ≥500 cells/μL (27.3%, 36/132), and <200 cells/μL (18.2%, 24/132). The most common initial CD8 T cell counts were ≤1000 cells/μL (38.6%, 51/132), 1001–1999 cells/μL (46.2%, 61/132), and ≥2000 cells/μL (15.2%, 20/132). Moreover, the mean ± standard deviation of CD4/CD8 was 0.32±0.19. The clinical symptoms of these 132 participants were grouped into four phases. Clinical phase II symptoms were the most frequent (75.8%, 100/132), followed by phase III (9.8%, 13/132), phase I (7.6%, 10/132), and phase IV (6.8%, 9/132) symptoms. The initial VLs of all participants were above three log RNA copies/mL (mean ± standard deviation 4.98±0.82 log RNA copies/mL). Most participants (87.1%, 115/132) had initial VLs ≥ 4 log RNA copies/mL, while 7.6% (10/132) of participants had initial VLs above 6 log RNA copies/mL.

**Table 1:** Demographic characteristics and distribution of HIV-1 subtypes among study participants.

Demographic characteristics	Cases	Subtypes				*DR*	χ^2^值	P值
		01_AE	07_BC	B	URFs				Other ^a^
Total	132	70	35	18	3	6	10		
Marital Status								5.574 ^b^	0.062
Married	75	40	17	11	1	6	2	17.149 ^c^	0.029
Unmarried	41	25	12	4	0	0	5		
Divorced/widowed	16	5	6	3	2	0	3		
Gender								0.993	0.319
Male	120	65	30	16	3	6	10	2.453	0.653
Female	12	5	5	2	0	0	0		
Age								0.076	0.963
<18	1	0	1	0	0	0	0	11.461	0.017
18–49	105	60	25	13	1	6	8		
≥50	26	10	9	5	2	0	2		
Infection route								1.764	0.414
MSM	112	60	27	16	3	6	10	7.232	0.512
HET	18	10	7	1	0	0	0		
Other ^d^	2	0	1	1	0	0	0		
First CD4 cell count (cells/mm^3^)								3.344	0.188
<200	24	17	4	3	0	0	4	9.402	0.309
200~499	72	34	19	11	2	6	5		
≥500	36	19	12	4	1	0	1		
First CD8 cell count (cells/mm^3^)								2.796	0.247
≤1000	51	30	12	7	0	2	3	6.638	0.576
1001~1999	61	29	16	9	3	4	7		
≥2000	20	11	7	2	0	0	0		
CD4/CD8								1.003	0.606
< 0.30	71	40	18	9	2	2	7	1.908	0.753
0.30~0.50	38	16	12	5	1	4	2		
≧0.50 ^e^	23	14	5	4	0	0	1		
First HIV VL (log RNA copies/ml)								2.823	0.420
3~	17	7	8	2	0	0	3	12.815	0.383
4~	55	29	14	8	3	1	4		
5~	50	28	12	6	0	4	2		
≥6	10	6	1	2	0	1	1		
Clinical phase								8.808	0.032
I	10	5	4	0	0	1	0	10.169	0.601
II	100	50	27	15	3	5	6		
III	13	7	3	3	0	0	4		
IV	9	8	1	0	0	0	0		

^a^Other subtypes included CRF55_01B (two individuals), CRF103_01B (two individuals), CRF65_cpx (one individual), and C (one individual).

^b^Result of χ^2^-tests comparing distributions of HIV-1 PDR in individuals with different demographic characteristics.

^c^Result of χ^2^-test comparing the distributions of HIV-1 subtypes in individuals with different demographic characteristic.

^d^Blood transmission (one participant) and mother-to-child transmission (one participant).

HIV-1, human immunodeficiency virus type 1; VL, viral load; HET, heterosexual; MSM, men who have sex with men; URF, unique recombinant form; CRF, circulating recombinant form.

^e^The highest value of CD4/CD8 is 0.91 among these study subjects.

### HIV-1 subtypes circulating in Baoding

**Fig 2:**
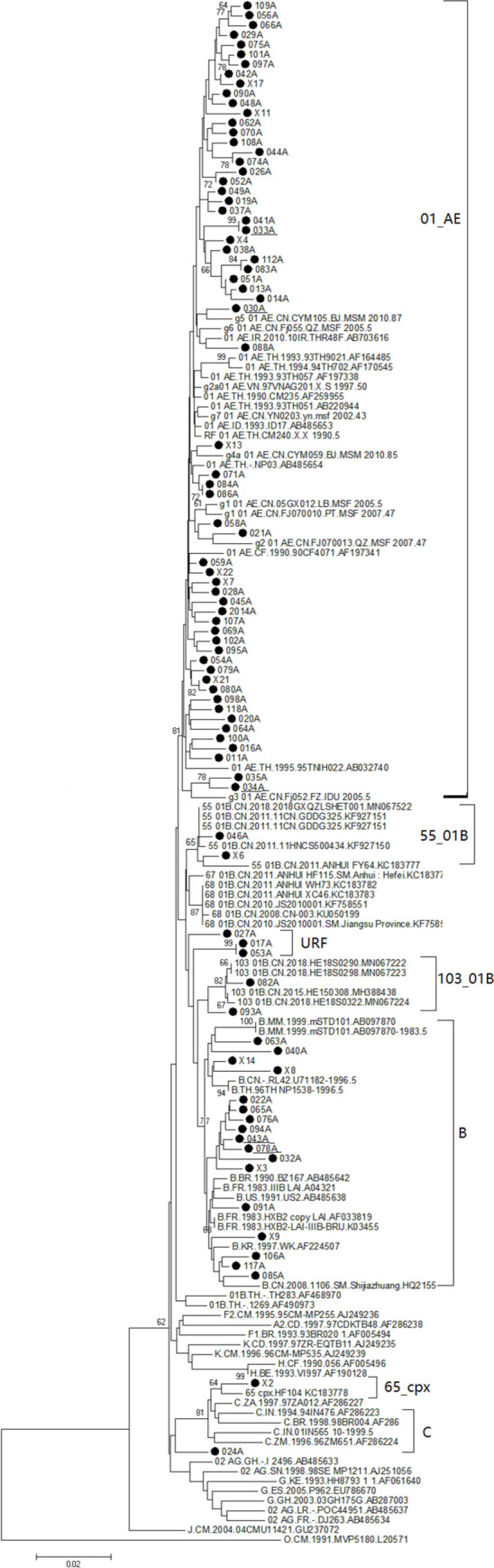
Phylogenetic tree based on partial *pol* gene sequences from all HIV-1 subtypes identified in this study except for CRF07_BC. The neighbor-joining tree was constructed using MEGA 6.0 with 1000 bootstrap replicates. The reference sequences (A–D, F–H, J, K, O, CRF01_AE, and others) were obtained from the HIV database (http://www.hiv.lanl.gov/content/index). Bootstrap values ≥60% are shown in the tree. The scale length indicates 2% nucleotide sequence divergence. Black dots denote sequences determined in this study.

**Fig 3:**
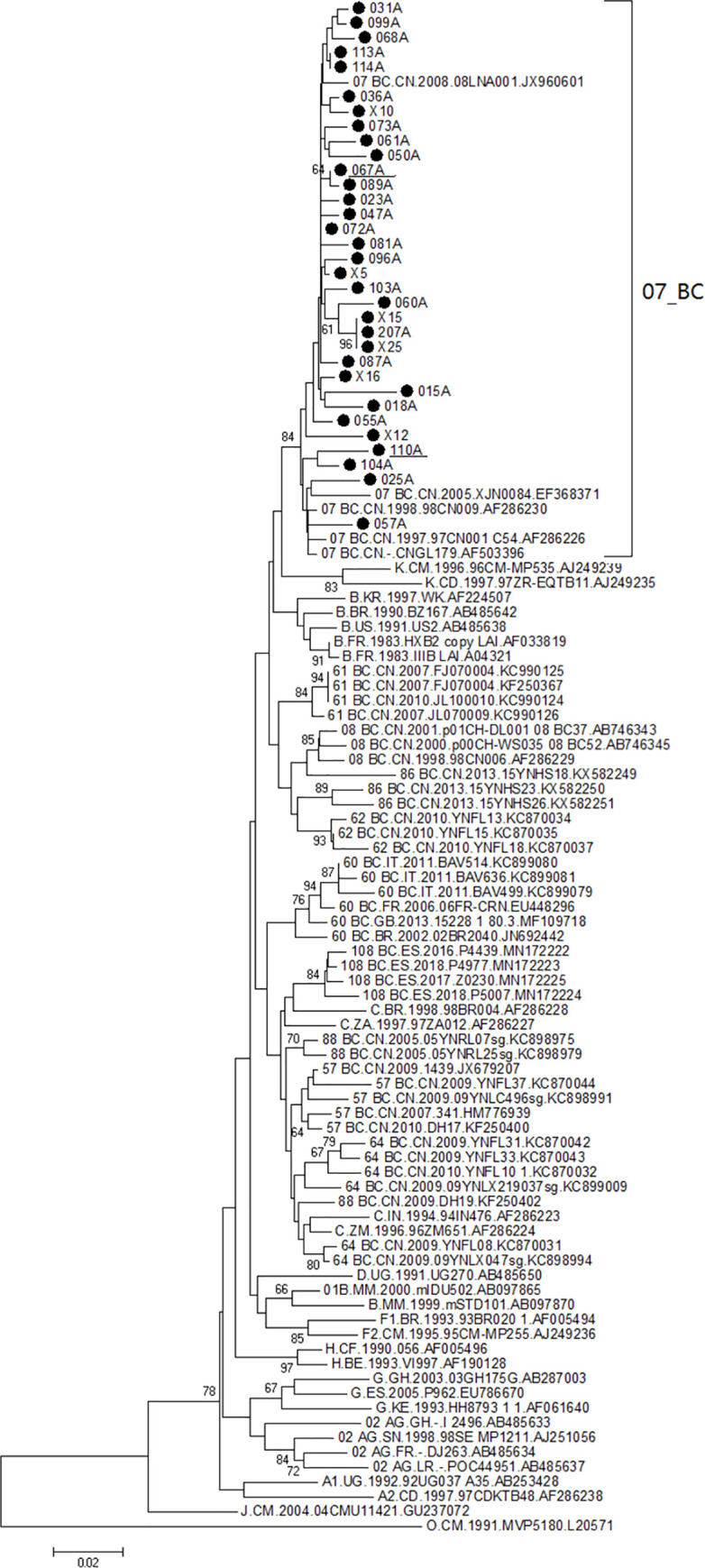
Phylogenetic tree based on partial *pol* gene sequences from HIV-1 CRF07_BC. The neighbor-joining tree was constructed using MEGA 6.0 with 1000 bootstrap replicates. The reference sequences (A–D, F–H, J, K, O, CRF07_BC and others) were obtained from the HIV database (http://www.hiv.lanl.gov/content/index). Bootstrap values ≥60% are shown in the tree. The scale length indicates 2% nucleotide sequence divergence. Black dots denote sequences determined in this study.

As shown in Figs 2 and 3, eight HIV-1 subtypes were detected in this study. The most frequent subtype was circulating recombinant form (CRF)01_AE (53.0%, 70/132), followed by CRF07_BC (26.5%, 35/132), B (13.6%, 18/132), unique recombinant forms (URFs) (2.3%, 3/132), CRF55_01B (1.5%, 2/132), CRF103_01B (1.5%, 2/132), CRF65_cpx (0.8%, 1/132), and C (0.8%, 1/132). Among participants with acute HIV-1 infection, recombinant strains accounted for 75% of isolates (6/8).

Table 1 shows HIV-1 subtype distributions. All eight subtypes were circulating in participants with the following characteristics: male, married, age 18–49 years, initial CD4 T cell count 200–499 cells/μL, initial CD8 T cell count 1001–1999 cells/μL, VL 4–5 log RNA copies/mL, clinical phase II symptoms, and MSM transmission. χ2-tests indicated differences in the distribution of HIV-1 subtypes by marital status (P<0.05) and age (P<0.05). However, HIV-1 subtype distribution was not significantly associated with gender, transmission route, initial CD4 T cell count, initial CD8 T cell count, or initial VL (Table 1). The distributions of CRF01_AE, CRF07_BC, and subtype B HIV-1 showed statistically significant associations (P<0.05) with all demographic characteristics. These subtypes were detected in participants of all demographic groups with the following exceptions: CRF01_AE was not detected in participants aged <18 years or in cases of bloodborne or mother-to-child transmission; and subtype B was not detected in participants aged <18 years or in those with clinical phase I or clinical phase IV symptoms. All HIV-1 URFs were detected in participants with the following characteristics: male, age ≥18 years, initial CD4 T cell count ≥200 cells/μL, initial CD8 T cell count 1001–1999 cells/μL, CD4/CD8 < 0.50, initial VL 4–5 log RNA copies/mL, clinical phase II symptoms, and MSM transmission.

### Distribution of HIV-1 PDR mutations

Among the 132 participants, 24.2% (32/132) had HIV-1 *pol* gene mutations. The locations of mutations are listed in Table 2 and including positions 46, 50, 53, 74, 48, 58, 32, 47, and 43 in the PI coding region; positions 75 and 151 in the NRTI coding region; and positions 101, 106, 138 and 179 in the NNRTI coding region. The most frequent mutations were V106I (12.1%, 16/132), followed by V179E/D/VE (7.6%, 10/132), E138A/EG/EK (3.8%, 5/132), M46I/L/MLRW (3.8%, 5/132), Q58E/QE (3.0%, 4/132), V32VA/VE/VG (3.0%, 4/132), K101E/KE (2.3%, 3/132), and other mutations (all <2.0%). Mutations clearly associated with PDR only accounted for 26.7% (4/15) of mutations. These included I50IFLM, Q151QL, K101E/KE, and E138A/EG/EK causing high-level resistance to atazanavir (ATV/r), intermediate-level resistance to abacavir and azidothymidine, low- to intermediate- resistance to doravirine, efavirenz (EFV), etravirine (ETR), nevirapine (NVP), and rilpivirine (RPV), and low-level resistance to RPV, respectively. Overall, 7.6% (10/132) of sequences showed clear resistance to ART. The prevalence of PDR to NNRTIs, NRTIs, and PIs was 6.1% (8/132), 0.8% (1/132), and 0.8% (1/132), respectively. Although the V179D/E and V106I substitutions in isolation do not lead to PDR, they can increase resistance associated with other mutations when they occur together. The occurrence of V106I together with V179E (sample ID: 043A) caused low-level PDR to ETR, NVP, and RPV. The E138EG mutation only leads to low-level PDR to RPV. However, the occurrence of E138EG together with V179D (sample ID: 033A) caused low-level PDR to EFV, ETR, NVP, and RPV, and the occurrence of E138EG together with V106I (sample ID: 078A) caused low-level PDR to ETR, NVP, and RPV.

**Table 2:** HIV-1 PDR mutations among 132 participants.

DR mutations	Participants containing mutation site	Frequency (%)	Subtypes	Infection routes	DR to antiretroviral drugs		
					PIs	NRTIs	NNRTIs
			(N)	(N)			
PI Major Mutations							
M46I/L/MLRW	5	3.8	01_ AE(4),B(1)	MSM(5)	-		
I50IFLM	1	0.8	01_ AE(1)	MSM(1)	ATV/r(H)		
PI Accessory Mutations							
F53FL	2	1.5	01_ AE(1),07_BC(1)	MSM(2)	-		
T74TP	1	0.8	01_ AE(1)	MSM(1)	-		
G48GR/GW	2	1.5	01_ AE(1),07_BC(1)	MSM(1),MTCT(1)	-		
Q58E/QE	4	3.0	01_ AE(3),07_BC(1)	MSM(4)	-		
V32VA/VE/VG	4	3.0	01_ AE(3),07_BC(1)	MSM(4)	-		
I47IR	1	0.8	01_ AE(1)	MSM(1)	-		
K43KT	1	0.8	01_ AE(1)	MSM(1)	-		
NRTI Mutations							
Q151QL	1	0.8	01_ AE(1)	MSM(1)		ABC(I)AZT(I)	
V75I	1	0.8	01_ AE(1)	MSM(1)		-	
NNRTI Mutations							
K101E/KE	3	2.3	01_AE(2),07_BC(1)	MSM(3)			DOR(L)EFV(L)ETR(L)NVP(I)RPV(I)
E138A/EG/EK	5	3.8	01_AE(3),07_BC(1),B(1)	MSM(5)			RPV(L)
V106I	16	12.1	01_AE(3), 103_01B (1), B(11), URF(1)	MSM(15),BT(1)			-
V179E/D/VE	10	7.6	01_AE(6),55_01BC(2),B(1) 65_cpx(1)	MSM(9),HET(1)			-

HIV-1, human immunodeficiency virus type 1; PDR, pre-treatment drug resistance; PI, protease inhibitor; NRTI, nucleoside reverse transcriptase inhibitor; NNRTI, non-nucleoside reverse transcriptase inhibitor; MSM, men who have sex with men; BT, blood transmission; MTCT, mother-to-child transmission; HET, heterosexual; -, no drug resistance; H, high-level resistance; I, intermediate resistance; L, low-level resistance; URF, unique recombinant form. ATV/r atazanavir/r; ABC abacavir; AZT zidovudine; EFV efavirenz; ETR etravirine; NVP, nevirapine; RPV, Rilpivirine; DOR, doravirine.

The distribution of HIV-1 PDR was not significantly associated with gender, marital status, age, transmission route, initial CD4 T cell count, initial CD8 T cell count, or initial VL (Table 2). However, the distributions of HIV-1 PDR differed significantly by clinical phase (P<0.05). Following stratification by clinical phase, the prevalence of HIV-1 PDR among participants with clinical phase III symptoms (30.8%, 4/13) was higher than that among participants with clinical phase II symptoms (6.0%, 6/100). Among participants with clinical phase I and clinical phase IV symptoms, no HIV-1 PDR was detected. The geographic distribution of HIV-1 PDR is shown in Fig 1. HIV-1 strains with PDR mutations were circulating in eight counties concentrated in the central and eastern regions of Baoding: Lianchi, Jingxiu, Qingyuan, Shunping, Yixian, Anxin, Gaoyang, and Rongcheng. These counties are geographically adjacent to one another except for Yixian. This finding suggested that despite the concentration of HIV-1 strains with PDR, there is a high risk of outward spread.

### HIV-1 molecular transmission networks

**Fig 4:**
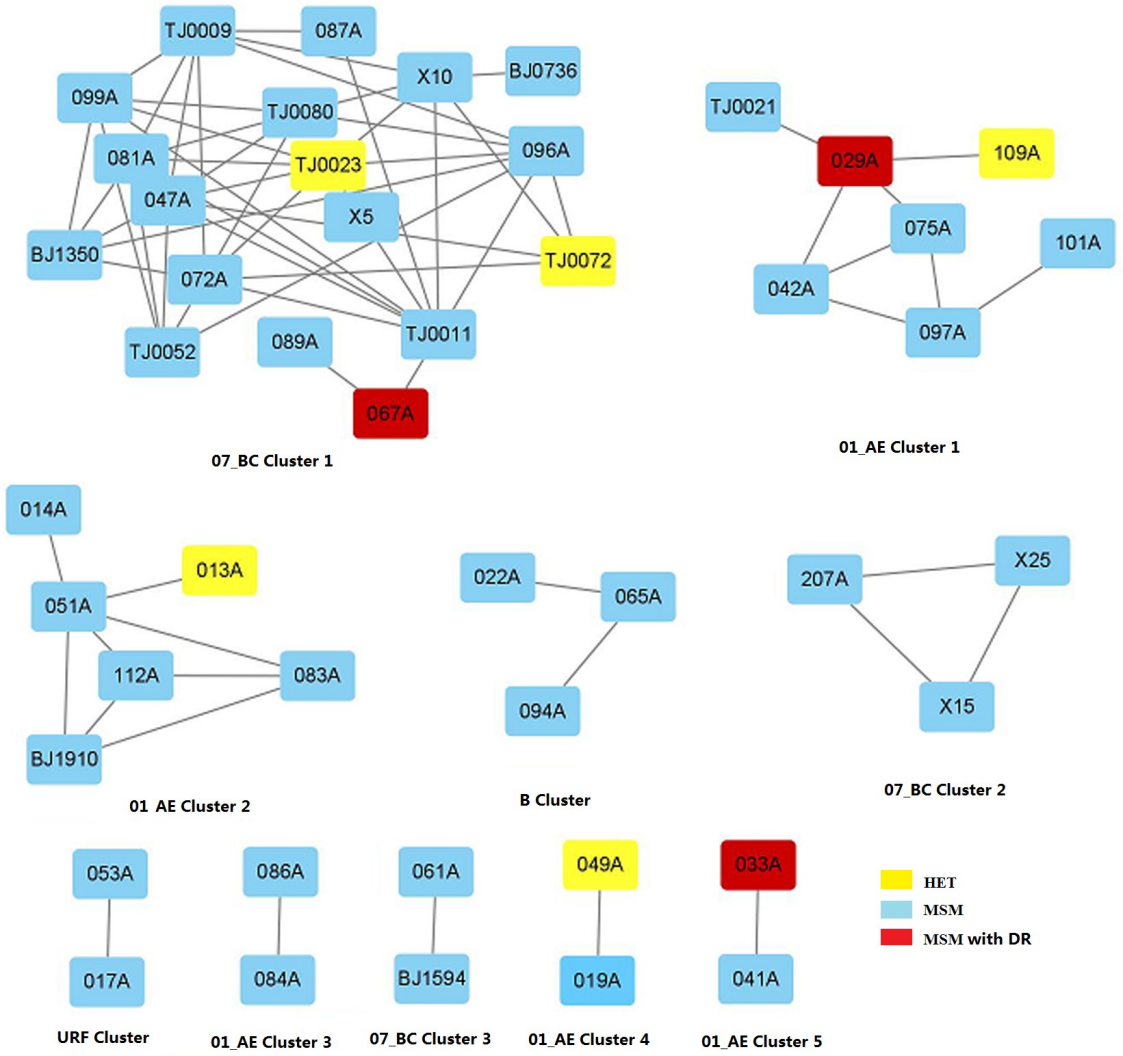
HIV-1 molecular transmission networks. TJ, Tianjin; BJ, Beijing; DR, drug resistance; HET, heterosexual; MSM, men who have sex with men.

To construct HIV-1 transmission networks, a total of 334 partial HIV-1 *pol* gene sequences (1.3 kb) were analyzed including 132 sequences determined in this study, 160 sequences from Beijing, and 42 sequences from Tianjin. Construction of HIV-1 molecular transmission networks with a genetic distance threshold of 0.015 identified the maximum number of clusters. Subsequently, 47 of 334 (14.0%) *pol* gene sequences were used to generate 10 molecular transmission clusters (Fig 4). Half (5/10) of clusters contained three or more participants. Among 47 participants who were included in these networks, five were infected via HET transmission and 42 were infected via MSM transmission. HIV-1 molecular transmission networks included five CRF01_AE clusters, three CRF07_BC clusters, one B cluster, and one URF cluster. CRF07_BC cluster 1, CRF01_AE cluster 1, and CRF01_AE cluster 2 showed that the sequences determined in this study were closely related with sequences from Beijing and Tianjin. Both MSM and HET transmission events were included in these three clusters.

As shown in Figs 1 and 4, CRF07_BC cluster 1 was an intricate transmission network consisting of HIV-1-infected individuals in Baoding (10 MSM), Beijing (two MSM), and Tianjin (two heterosexuals and four MSM). In CRF07_BC cluster 1, sequences were obtained in this study from geographically dispersed counties in Baoding. Nearly all sequences had more than four degrees except for 067A, 087A, 089A and BJ0736 in CRF07_BC cluster 1, and sequences from Tianjin even had nine degrees. However, the degrees were below three in the remaining clusters. CRF01_AE clusters 1 and 2 were related to Tianjin and Beijing, respectively. Geographically, study sequences in CRF01_AE cluster 1 were distributed in Gaoyang, Laiyuan, Lianchi, Dingzhou, and Xushui. By contrast, study sequences in CRF01_AE cluster 2 were concentrated in Mancheng, Jingxiu, Anxin, and Lianchi. Study sequences in the remaining clusters (≤ 3 sequences each) were concentrated in either one county or neighboring counties, except for the subtype B cluster containing sequences from Dingzhou and Yixian.

Sequences from three of five heterosexuals involved in molecular transmission networks were obtained in the current study (109A in CRF01_AE cluster 1, 013A in CRF01_AE cluster 2, and 049A in CRF01_AE cluster 5). Fig 4 shows that participants 109A, 013A, and 049A had simple transmission relationships within their respective networks and were only related to participants 029A, 051A and 019A, respectively. However, participants 029A and 051A were closely associated with more than three MSM. Participant 013A (aged 21 years) and participant 051A (aged 27 years) were from Lianchi and Mancheng, respectively; participant 019A (aged 59 years) and participant 049A (aged 46 years) were from Dingzhou and Anguo, respectively; and participant 029A (aged 29 years) and participant 109A (aged 26 years) were from Gaoyang and Xushui, respectively. Two of these three pairs (participants 019A and 049A and participants 029A and 109A) were in non-marital heterosexual contact according to face-to-face interviews. Participant 013A did not have a sexual history with participant 051A, suggesting that a third person who was not included in this study played a key role in transmission between 013A and 051A.

In this study, three of ten sequences associated with PDR were included in the transmission networks (Fig 4). These three sequences were found in the largest cluster (CRF07_BC cluster 1), the second largest cluster (CRF01_AE cluster 1), and the smallest cluster (CRF01_AE cluster 5) and were detected in Yixian (participant 067A), Gaoyang (participant 029A), and Anxin (participant 033A), respectively. CRF07_BC cluster 1 contained one MSM whose virus contained a mutation associated with PDR to NNRTIs (K101KE); CRF01_AE cluster 1 contained one MSM whose virus contained mutations associated with PDR to PIs (M46MLRW, I50IFLM, F53FL, T74TP); and CRF01_AE cluster 5 contained one MSM whose virus contained mutations associated with PDR to NNRTIs (E138EG,V179D). No transmission of HIV-1 with PDR mutations was observed among the remaining five clusters. No mutations conferring PDR to NRTIs were found in molecular transmission networks.

## Discussion

In this study, HIV-1 genetics were analyzed among newly diagnosed drug-naïve patients during acute HIV-1 infection in Baoding City, Hebei Province. Eight HIV-1 subtypes were circulating in these individuals. Recombinant strains accounted for 75% (6/8) of these eight HIV-1 subtypes, including five CRFs and one URF. all HIV-1 subtypes identified were circulating in participants with the following characteristics: male, married, age 18–49 years, initial CD4 T cell count 200–499 cells/μL, initial CD8 T cell count 1001–1999 cells/μL, CD4/CD8 < 0.50, VL 4–5 log RNA copies/mL, clinical phase II symptoms, and MSM transmission. This suggests that older individuals have become a high-risk population for HIV-1 spread. Strikingly, participants infected with all HIV-1 subtypes were immunodeficient, and high HIV-1 VLs, had clinical phase II symptoms, were married, and were infected via MSM transmission. Married individuals with acute HIV-1 infection involved in MSM transmission can bidirectionally transmit the virus through homosexual or heterosexual contacts. Individuals aged 18–49 years accounted for 79.5% of all participants and were infected with almost all HIV-1 subtypes except for URFs. In Hebei, annual numbers of HIV-1 cases among older individuals as well as among youths are continuing to increase. Therefore, HIV-1 surveillance efforts among youths and elderly people should be strengthened. Additionally, HIV-1 subtype distributions were not associated with demographic characteristics except for marital status (P<0.05) and age (P<0.05). Furthermore, the most common three HIV-1 subtypes were distributed in participants of almost all demographic characteristics, suggesting that HIV-1 subtypes circulating in Baoding have spread into the general population.

The highest numbers of HIV-1 subtypes were circulating in married participants (eight subtypes), followed by divorced/widowed participants (four subtypes) and unmarried participants (three subtypes). The highest numbers of HIV-1 subtypes were circulating in participants aged 18–49 years (eight subtypes), participants aged ≥50 years (four subtypes), and participants aged <18 years (one subtype). HIV-1 subtype distribution was significantly associated with marital status and age. We infer that marital status and age are closely associated with changes in MSM sexual behaviors, and that sexual contact patterns play a key role in these differences.

Some studies [18, 19] reported that genetic barriers for PDR to NNRTIs were low because of their long plasma half-lives and the fact that a single point mutation in the NNRTI coding region can cause PDR. Therefore, the prevalence of PDR to NNRTIs is used as a criterion for initiating public health responses by the WHO [20]. The WHO`s 2019 report on HIV-1 drug resistance [1] indicated a high prevalence (≥10%) of PDR to EFV and/or NVP among adults initiating first-line ART in 12 of 18 low- and middle-income countries. PDR to NNRTIs showed an increasing trend. In China, NNRTI-based ART regimens (including NVP and EFV) have been extensively used for first line therapy since the initiation of free ART in 2003 [10]. In this study, the prevalence of PDR was 7.6% among participants with acute HIV-1 infection in Baoding. The prevalence of PDR in Baoding from 2019–2020 was moderate (5–15%) according to the WHO definition. This prevalence of PDR was higher than that in Hebei (6.1%) [21], Beijing (4.1%) [22], China overall (6.8%) [10] and in other areas [12, 23], Tianjin (11.5%), Zimbabwe (6.3%), South/Southeast Asia (2.9%), upper-income Asian countries (5.6%), and Latin America/Caribbean (7.6%), but lower than that in Cameroon (9.8%), Europe (9.4%), and North-America (11.5%). Moreover, 6.1% of subjects carried NNRTI resistance-related mutations such as K101, E138, V106 and V179. Although there was a moderate prevalence of PDR in Baoding, these data demonstrate a high risk of PDR introduction into the general population via sexual contact because of the high VLs associated with acute HIV-1 infection. ATV/r has never been used in ART regimens in Hebei. However, one participant’s virus carried PI-associated mutations (M46MLRW, I50IFLM, F53FL, T74TP) that confer high-level PDR to ATV/r. This participant was involved in the second largest transmission network (CRF01_AE Cluster 1, Fig 4). This finding suggests that ATV/r-resistant HIV-1 strains in Baoding may originate from other provinces (for example, Tianjin). In key populations, the prevalence of PDR was high [9] and exceeded 10% among MSM, sex workers, and prisoners. MSM were found to have a higher prevalence of PDR compared with injection drug users and the general population. In the current study, 84.8% of participants were MSM and harbored all viruses with PDR mutations. The risks of spreading resistant HIV-1 strains via transmission networks are significantly increased during acute HIV-1 infection.

To explore HIV-1 transmission in individuals with acute HIV-1 infection and whether HIV-1 PDR was circulating in this population before initiation of ART, we constructed HIV-1 molecular transmission networks. In our study, a total of 10 transmission networks were identified. The CRF01_AE and CRF07_BC transmission clusters accounted for 50% and 30% as the most frequent two HIV-1 subtypes, respectively, and were closely associated with Beijing and Tianjin. This finding suggests that CRF01_AE and CRF07_BC will remain the most common HIV-1 strains in the next few years.

We identified three HIV-1 molecular transmission networks each containing a different HIV-1 PDR mutation (Fig 4): the largest transmission network (CRF07_BC cluster 1), the second largest transmission network (CRF01_AE cluster 1), and CRF01_AE cluster 5; these clusters contained a single NNRTI mutation (K101KE), multiple PI mutations (M46MLRW, I50IFLM, F53FL and T74TP), and two NNRTI mutations (E138EG and V179D), respectively. The largest transmission network contained participants from Tianjin and Beijing; most participants, including two heterosexuals and two MSM, were from Tianjin. The second largest transmission network contained one participant from Tianjin; this participant had only one contact in the network but he was closely connected with a participant whose virus bore mutations associated with PDR to PIs. Another transmission network contained only two MSMs. Both Beijing and Tianjin are large cosmopolitan cities that are busy traffic hubs with large transient populations from other provinces in China and other countries. Regional variation in the prevalence of HIV-1 strains has been mainly attributed to differences in HIV-1 transmission modes. In southwestern provinces, HIV-1 transmission has seen a shift from intravenous injection to HET transmission; however, MSM has become the most frequent transmission route in the Beijing-Tianjin-Hebei region. The prevalence of HIV-1 subtypes in Hebei was consistent with those in Beijing and Tianjin. Our results suggest that HIV-1 PDR mutations could be spread via transmission networks consisting of high-risk populations in Hebei, Tianjin and Beijing. Resistant strains could also be spread to the general population via heterosexual contact.

The present work had two limitations. First, Hebei has four (Baoding, Langfang, Zhangjiakou, and Chengde) of 11 cities which shares a border with Beijing. This study can`t represent all individuals living with HIV-1 in all four cities. Second, although HIV-1 transmission among individuals with acute HIV-1 infection were investigated, the study results can`t adequately reflect the situation of HIV-1 spread in the whole city and the transmission relationships between neighboring provinces and Baoding of Hebei due to the lack of individuals with non—acute HIV-1 infection. Later, We will expand the further study to all individuals living with HIV-1 in all four cities in Hebei, sharing a border with Beijing.

## Conclusions

This study represents the first investigation of HIV-1 genetics and transmission networks among key populations with acute HIV-1 infection in Hebei, a city sharing a border with Beijing and Tianjin. Our results demonstrate that during acute infection, HIV-1 genetic diversity is complex and evolving and that HIV-1 strains are circulating in transmission networks consisting of participants from Beijing, Tianjin, and Hebei. A moderate prevalence of HIV-1 PDR was observed in Baoding (7.6%); HIV-1 strains bearing PDR mutations were detected in major networks. These data suggest that it is critical to ensure early detection, diagnosis, surveillance, and treatment to effectively control HIV-1 spread.

## Supporting information

S1 Checklist STROBE statement—Checklist of items that should be included in reports of observational studies. (DOCX)

## References

[1] UNAIDS. 2021 Progress Report on the Global AIDS response. Available from: https://www.sohu.com/a/477822682_121106902.

[2] Tomas C and Marshall F. Current status and prospects of HIV treatment. Current Opinion in Virology. 2016; 18:50–56. Available from: www.sciencedirect.com. doi: 10.1016/j.coviro.2016.03.004 27023283

[3] Francesco RS, Mary FK. Review: Influence of ART on HIV genetics. Curr Opin HIV AIDS. 2015; 10(1):49–54. doi: 10.1097/COH.0000000000000120 25389802 PMC4392820

[4] Chen S, Ma L, Lu X, Li Y, Wang W, Wang Y, et al. Effect of highly active anti-retroviral therapy on reducing HIV/AIDS related death in Hebei, 1989–2013. Chin J Epidemiol. 2015; 36(5): 460–464. 26080634

[5] Lu DY, Wu HY, Yarla NS, Xu B, Ding J, Lu TR. HAART in HIV/AIDS treatments: future trends. Infectious Disorders-Drug Targets. 2018; 18(1): 15–22. doi: 10.2174/1871526517666170505122800 28474549

[6] UNAIDS. 90-90-90 An Ambitious Treatment Target to Help End the AIDS Epidemic. Available from: http://www.unaids.org/sites/default/files/media_asset/90-90-90_en_0.pdf (2014).

[7] Gupta RK, Gregson J, Parkin N, Hiwot H, Tanuri A, Forero L, et al. HIV-1 drug resistance before initiation orre-initiation of first-line antiretroviral therapy in low-income and middle-income countries: a systematic review and meta-regression analysis. Lancet Infect Dis. 2018; 18, 346–355. doi: 10.1016/S1473-3099(17)30702-8 29198909 PMC5835664

[8] World Health Organisation (WHO). HIV Drug Resistance Report 2019. Geneva, Switzerland: World Health Organization; 2019.

[9] Virginia M, Lawrence M, Michael RJ, Bradley M, Sharon J, Rachel B, et al. Prevalence of pretreatment HIV drug resistance in key populations: a systematic review and meta-analysis. Journal of the International AIDS Society. 2020; 23:e25656. doi: 10.1002/jia2.25656 33369131 PMC7758978

[10] Kang R, Liang S, Ma Y, Liang S, Xiao L, Zhang X, et al. Pretreatment HIV drug resistance in adults initiating antiretroviral therapy in China, 2017. Infect Dis Poverty. 2020; 9(1): 54. doi: 10.1186/s40249-020-00668-5 32448388 PMC7247188

[11] Wang Z, Zhang M, Zhang R, Liu L, Shen Y, Wang J, et al. Diversity of HIV-1 genotypes and high prevalence of pretreatment drug resistance in newly diagnosed HIV-infected patients in Shanghai, China. BMC Infectious Diseases. 2019; 19:313. Available from: doi: 10.1186/s12879-019-3927-1 30961560 PMC6454613

[12] Zeng R, Ren D, Gong X, Wei M, Gao L, Yu A, et al. HIV-1 Genetic Diversity and High Prevalence of Pretreatment Drug Resistance in Tianjin, China. AIDS Res Hum Retroviruses 2020, 36(10): 852–861. doi: 10.1089/AID.2020.0056 32539490

[13] Teng Xun Wang. A summary of HIV/AIDS epidemic situation across the country in 2020. Available from: https://xw.qq.com/amphtml/20201207A0B1EE00. 2020-12-07.

[14] Yan Zhao Evening News. HIV-infected cases in the two regions accounted for 37.1% of HIV infections found in all counties of the whole Hebei province. Available from: http://hebei.sina.com.cn/news/m/2016-11-30/detail-ifxyawmp0619417.shtml?bsh_bid=1577422709.

[15] AIDS and Hepatitis C Professional Group, Society of Infectious Diseases, Chinese Medical Association; Chinese Center for Disease Control and Prevention. Chinese guidelines for diagnosis and treatment of HIV/AIDS (2018). Chin J Intern Med. 2018; 57(12): 867–883. doi: 10.3760/cma.j.issn.0578-1426.2018.12.002 30486555

[16] Chinese Center for Disease Control and Prevention. Manual of the national free antiretroviral treatment. 4th edn. Beijing: People’s Medical Publishing House, 2016.

[17] Lu X, Zhao H, Zhang Y, Wang W, Zhao C, Li Y, et al. HIV-1 drug-resistant mutations and related risk factors among HIV-1-positive individuals experiencing treatment failure in Hebei Province, China. AIDS Res Ther. 2017; 14:4. doi: 10.1186/s12981-017-0133-3 28114955 PMC5260017

[18] Usach I, Melis V and Peris JE. Non-nucleoside reverse transcriptase inhibitors: a review on pharmacokinetics, pharmacodynamics, safety and tolerability. J Int AIDS Soc. 2013; 16(1): 1–14. doi: 10.7448/IAS.16.1.18567 24008177 PMC3764307

[19] Mackie N. Resistance to non-nucleoside reverse transcriptase inhibitors. In Geretti AM(ed.), Antiretroviral Resistance in Clinical Practice. London: Mediscript, 2006. Available from: https://www.ncbi.nlm.nih.gov/books/NBK2239/. 21249773

[20] World Health Organization. Update of recommendations on first- and second-line antiretroviral regimens Available from: https://www.who.int/hiv/pub/arv/arv-update-2019- policy /en/. Accessed July 2019.

[21] Lu X, Chen S, Zhao H, Li Y, Wang Y, Zhang Y, et al. Baseline investigation of HIV-1 primary drug resistance among newly diagnosed treatment-naïve HIV-1 individuals in Hebei, China. AIDS Research and Human Retroviruses. 2018; 34(12):1083–1089. doi: 10.1089/AID.2018.0142 29999406

[22] Ye J, Hao M, Xing H, Zhang F, Wu H, Lv W, et al. Transmitted HIV drug resistance among individuals with newly diagnosed HIV infection: a multicenter observational study. AIDS 2020, 34(4):609–619. doi: 10.1097/QAD.0000000000002468 31895143

[23] Rhee SY, Blanco JL, Jordan MR, Taylor J, Lemey P, Varghese V, et al. Geographic and Temporal Trends in the Molecular Epidemiology and Genetic Mechanisms of Transmitted HIV-1 Drug Resistance: An Individual-Patient- and Sequence Level Meta-Analysis. PLoS Med. 2015; 12(4):e1001810. Available from: doi: 10.1371/journal.pmed.1001810 25849352 PMC4388826

